# Effect of the coronavirus disease 2019 pandemic on paediatric bilateral myringotomy and tube insertion rates in New Brunswick, Canada

**DOI:** 10.1017/S002221512300066X

**Published:** 2023-12

**Authors:** K Hathi, C J Chin, B J A Hoyt

**Affiliations:** 1 Dalhousie Medicine New Brunswick, Saint John, Canada; 2 Division of Otolaryngology – Head and Neck Surgery, Department of Surgery, Dalhousie University, Halifax, Nova Scotia, Canada; 3 Department of Otolaryngology – Head and Neck Surgery, Horizon Health Network, New Brunswick, Canada

**Keywords:** Coronavirus, upper respiratory infections, public health, otitis media, otitis media with effusion

## Abstract

**Objective:**

To assess the effect of the coronavirus disease 2019 pandemic on paediatric bilateral myringotomy and tube insertion rates in New Brunswick, Canada.

**Methods:**

All paediatric bilateral myringotomy and tube insertion cases from 1 July 2015 through 30 June 2021 were provided by New Brunswick Medicare. The numbers of otolaryngologists, cataract surgical procedures, total hip arthroplasties and thyroidectomies were collected to assess the availability of operating theatres and otolaryngologists. Negative binomial logarithmic regressions were used for analyses.

**Results:**

Of the 5175 paediatric bilateral myringotomy and tube insertion cases that were included, the bilateral myringotomy and tube insertion rate significantly decreased by 2.9 times (*p* < 0.001) during the pandemic. Thyroidectomies, cataract surgical procedures and total hip arthroplasties did not significantly decrease. The number of otolaryngologists increased (20 *vs* 16–17).

**Conclusion:**

Paediatric bilateral myringotomy and tube insertion rates significantly decreased during the pandemic. This cannot be accounted for by reduced otolaryngologists or operating theatre availability. The paediatric bilateral myringotomy and tube insertion rate decrease is likely due to public health measures reducing the transmission of upper respiratory tract infections, resulting in fewer indications for paediatric bilateral myringotomy and tube insertion.

## Introduction

Viral transmission of the severe acute respiratory syndrome coronavirus 2 (SARS-CoV-2) caused the coronavirus disease 2019 (Covid-19) pandemic, which disrupted healthcare systems and daily life internationally.^1–3^ Due to this, governments implemented public health measures to reduce the viral transmission of Covid-19.^2,3^

The province of New Brunswick, Canada, responded quickly to the Covid-19 pandemic by announcing a public health state of emergency on 19 March 2020, 7 days after the first case in the province.^4,5^ Public health measures that were implemented in New Brunswick included cancelling non-urgent healthcare services, mandating physical distancing, limiting close contacts, and closing in-person schooling, day-care facilities, extracurricular activities and non-essential businesses.^4,5^ This resulted in New Brunswick having low Covid-19 transmission rates early in the pandemic. Restrictions began to ease through late May and early June 2020.^4,6^

Bilateral myringotomy and tube insertion is one of Canada's most common paediatric procedures.^7^ Common indications are recurrent acute otitis media with effusion, refractory acute otitis media, and chronic otitis media with effusion.^8^ Upper respiratory tract infections (URTIs) often contribute to the pathophysiology of these indications. Children typically are prone to URTIs due to their higher exposure to viral and bacterial pathogens at schools, day-care facilities and during extracurricular activities, as well as their developing immune systems.^9,10^

Anecdotally, there has been a decrease in the bilateral myringotomy and tube insertion rate since the onset of the Covid-19 pandemic. We hypothesise that public health measures aimed at reducing viral transmission may have resulted in fewer URTIs amongst children and, therefore, fewer indications to perform bilateral myringotomy and tube insertions.

The rate of bilateral myringotomy and tube insertions during the Covid-19 pandemic has not been studied in Canada's publicly funded healthcare system. The province of New Brunswick presents a unique setting to explore the effect of the Covid-19 pandemic because it had low Covid-19 case counts and strict public health measures early in the pandemic. This resulted in less strain on the provincial healthcare system and subsequently less disruption to the functioning of operating theatres. The effect of public health measures on bilateral myringotomy and tube insertion rates cannot be studied in a controlled setting given the challenges associated with randomising subjects to strict shelter-in-place and contact precautions. However, the Covid-19 pandemic offered the perfect natural experiment to study these effects. This study compared bilateral myringotomy and tube insertion rates in paediatric patients before and during the Covid-19 pandemic in the province of New Brunswick, Canada. The study used data from New Brunswick Medicare, which provided comprehensive results on all bilateral myringotomy and tube insertions performed in the province over six years.

## Materials and methods

### Data collection

This is a retrospective cohort study on the effect of Covid-19 public health measures on paediatric bilateral myringotomy and tube insertion rates. Approval for data collection and a waiver of consent were obtained from Horizon Health Network Research Ethics Board (ROMEO file number: 101442).

New Brunswick Medicare provided a dataset of all cases of bilateral myringotomy and tube insertions from 1 July 2015 through 30 June 2021, containing patient age, biological sex and date of procedure. Patients aged over 18 years were excluded from the dataset, leaving only paediatric patients. Adult patients were excluded because their indications for bilateral myringotomy and tube insertion are typically due to non-infectious aetiologies and, therefore, would be less likely to be affected by the Covid-19 pandemic and subsequent public health measures.

The number of providers per year that billed for a bilateral myringotomy and tube insertion from 2015 to 2021 was obtained from New Brunswick Medicare. These data ensured any observed differences were not due to a change in the number of providers.

The numbers of total hip arthroplasties, cataract surgical procedures and thyroidectomies (hemi- and total) during our study period were collected from New Brunswick Medicare in order to ensure that observed differences in bilateral myringotomy and tube insertion rates were not due to changes in the availability of operating theatres. These procedures were selected because: cataract surgery represents a benchmark for minor ambulatory procedures, total hip arthroplasty represents a major surgical procedure performed under general anaesthesia, and thyroidectomy is a higher urgency surgery within otolaryngology.

### Data analysis

Biological sex and patient age before and during the pandemic were described for the bilateral myringotomy and tube insertion cohort overall and for each individual. Categorical variables were presented as absolute counts and percentages, and continuous variables were presented as means ± standard deviations (SDs).

The number of bilateral myringotomy and tube insertions performed during each year, defined as 1 July through 30 June, was plotted with 95 per cent confidence intervals (CIs) calculated using Poisson distribution. The numbers of pre-pandemic (1 July 2015 through 29 February 2020) bilateral myringotomy and tube insertions, total hip arthroplasties, cataract surgical procedures and thyroidectomies per month were compared to the numbers of these same procedures per month during the pandemic (1 March 2020 through 30 June 2021) using negative binomial logarithmic regressions. The number of providers in New Brunswick that billed for a bilateral myringotomy and tube insertion per year was reported with 95 per cent CIs calculated with Poisson distributions.

## Results

**Figure 1.**
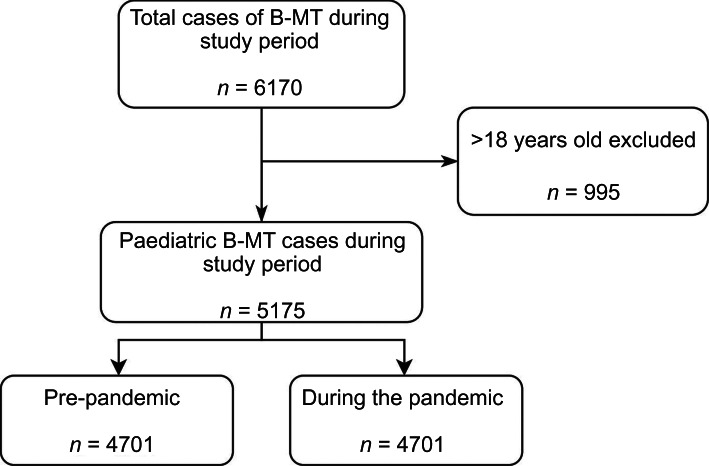
Flow chart of patient inclusions and exclusions. B-MT = bilateral myringotomy and tube insertion

Of the 6170 bilateral myringotomy and tube insertions performed from 1 July 2015 through 30 June 2021 in New Brunswick, 995 cases were excluded as the patients were aged over 18 years. In total, 5175 paediatric bilateral myringotomy and tube insertion cases were included in this study (Figure 1).

Patient demographics are listed in Table 1. The mean patient age was 4.8 ± 3.0 years; 39.3 per cent were female and 60.7 per cent were male. These values were similar both before and during the pandemic.

**Table 1.** Patient demographics for the bilateral myringotomy and tube insertions cohort*

Variable	Overall cohort*	Pre-pandemic^†^	During pandemic^‡^
Age (mean ± SD; years)	4.8 ± 3.0	4.7 ± 3.0	5.2 ± 3.4
Sex (*n* (%))			
– Females	2032 (39.3)	1840 (39.1)	192 (40.5)
– Males	3143 (60.7)	2861 (60.9)	282 (59.5)

*n = 5175; ^†^*n* = 4701; ^‡^*n* = 474. SD = standard deviation

**Figure 2.**
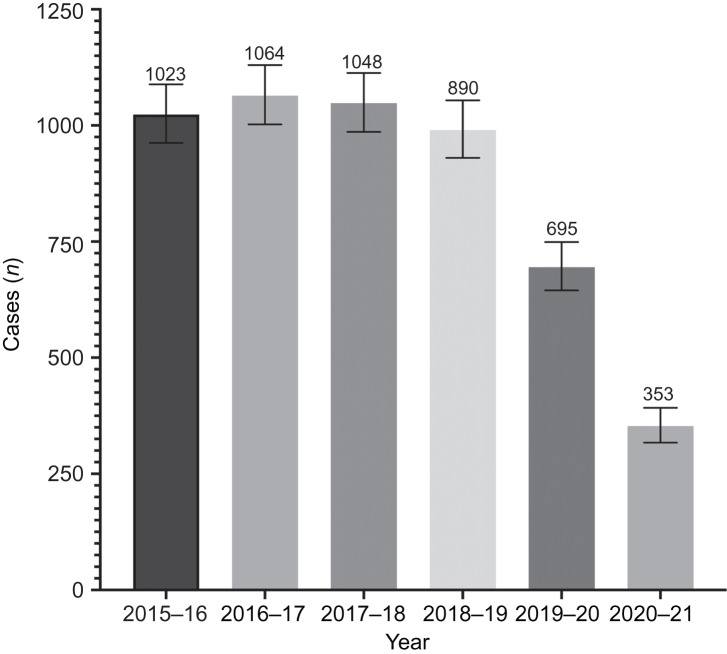
Bilateral myringotomy and tube insertion procedures per year. Error bars represent 95 per cent confidence intervals by Poisson distribution.

The numbers of pre-pandemic (2015–2019) bilateral myringotomy and tube insertions per year (1 July to 30 June) ranged from 695 to 1064 (95 per cent CI = 530–1130) (Figure 2). The number of bilateral myringotomy and tube insertions during the pandemic (1 July 2020 to 30 June 2021) was 353 (95 per cent CI = 317–392) (Figure 2). No bilateral myringotomy and tube insertion procedures were performed in New Brunswick from 25 March 2020 to 11 May 2020.

**Figure 3.**
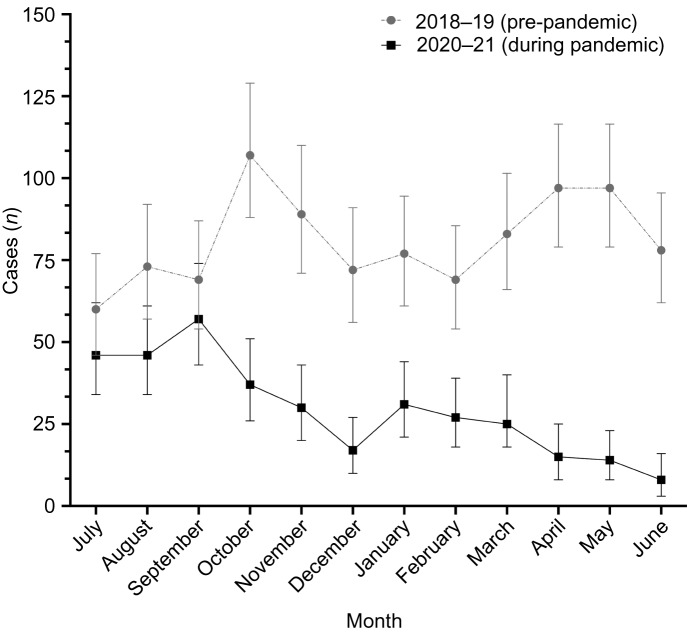
Bilateral myringotomy and tube insertion procedures in 2018–2019 and 2020–2021. Error bars represent 95 per cent confidence interval by Poisson distribution.

Figure 3 shows the numbers of bilateral myringotomy and tube insertions per month in 2018–2019 and in 2020–2021. A comparison of 2020–2021 with 2018–2019 was made because 2018–2019 is the most recent year not affected by the pandemic. The numbers of bilateral myringotomy and tube insertions per month in 2018–2019 (pre-pandemic) ranged from 60 to 107. Bilateral myringotomy and tube insertions per month in 2020–2021 (during the pandemic) ranged from 8 to 57. The lowest number of bilateral myringotomy and tube insertions per month in 2020–2021 was eight, which occurred during June 2021. The average number of bilateral myringotomy and tube insertions in 2018–2019 (pre-pandemic) was 990 (95 per cent CI = 930–1054), compared to an average of 353 bilateral myringotomy and tube insertions in 2020–2021 (during pandemic) (95 per cent CI = 317–392), which is a 64.3 per cent decrease of bilateral myringotomy and tube insertions from the pre-pandemic value.

**Figure 4.**
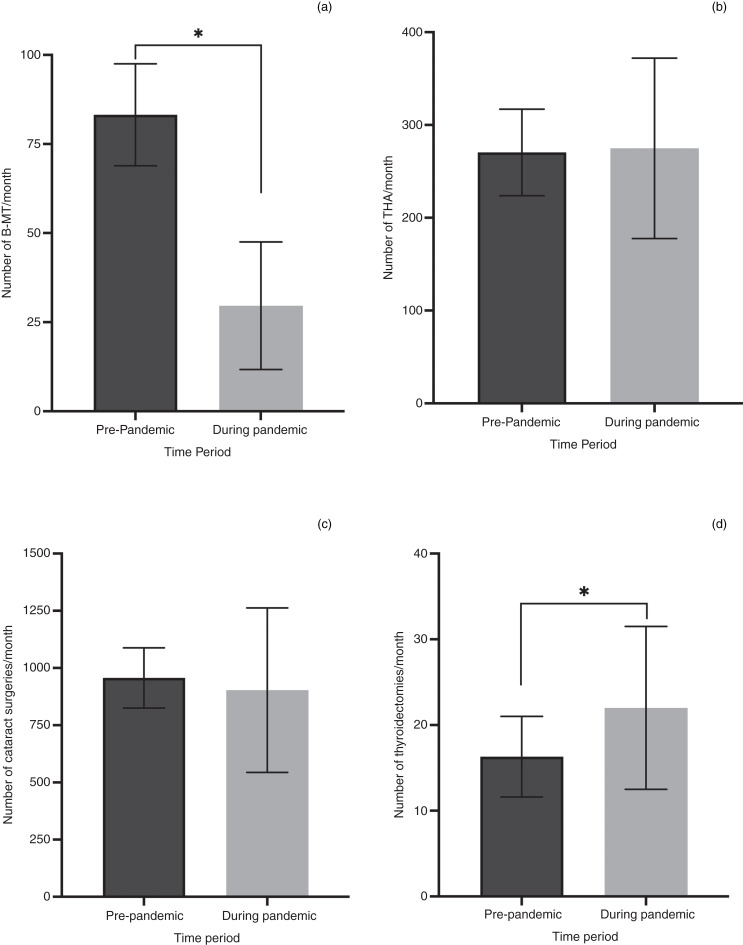
Mean number of procedures per month, before and during the pandemic. Error bars represent standard deviation (SD). *Indicates *p* < 0.05, based on negative binomial logarithmic regressions. B-MT = bilateral myringotomy and tube insertion; THA = total hip arthroplasty

The mean numbers of bilateral myringotomy and tube insertions, total hip arthroplasties, cataract surgical procedures and thyroidectomies performed in New Brunswick per month pre-pandemic and during the pandemic are represented in Figure 4a–d. The negative binomial logarithmic regression found that the average number of bilateral myringotomy and tube insertions per month significantly decreased by 2.9 times during the pandemic compared to the pre-pandemic average (*p* < 0.001, odds ratio = 2.90, 95 per cent CI = 2.42–3.48). No significant difference was found in the average number of total hip arthroplasty procedures or cataract surgical procedures per month during the pandemic compared to pre-pandemic (total hip arthroplasty *p* = 0.561, odds ratio = 0.96, 95 per cent CI = 0.82–1.12; cataract *p* = 0.871, odds ratio = 1.02, 95 per cent CI = 0.82–1.26). The average number of thyroidectomies per month significantly increased during the pandemic compared to pre-pandemic (*p* < 0.001, odds ratio = 0.71, 95 per cent CI = 0.60–0.85).

**Figure 5.**
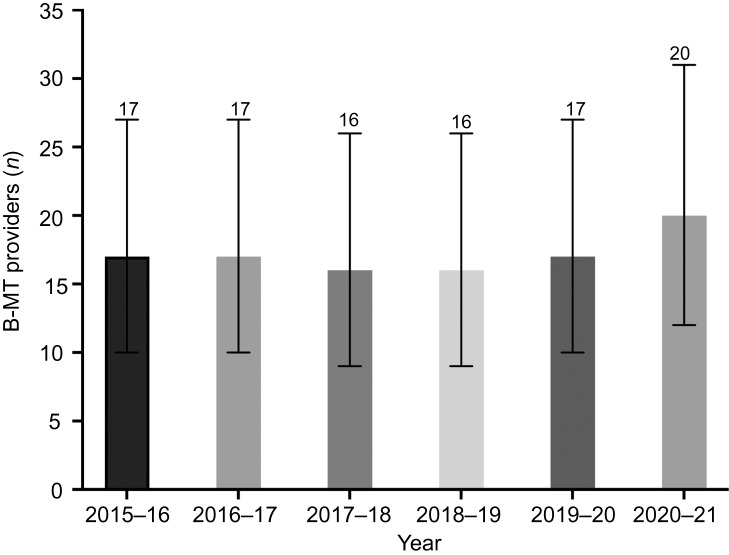
Number of paediatric bilateral myringotomy and tube insertion (B-MT) providers in New Brunswick per year. Error bars represent 95 per cent confidence interval by Poisson distribution. *Indicates significant differences in the number of procedures performed pre-pandemic versus during the pandemic

The number of providers that billed New Brunswick Medicare for a bilateral myringotomy and tube insertions per year pre-pandemic ranged from 16 to 17 (95 per cent CI = 9–27), while this number during the pandemic (2020–2021) was higher at 20 (95 per cent CI = 12–31) (Figure 5).

## Discussion

This study was a natural experiment on the effect of the Covid-19 pandemic and public health measures on paediatric bilateral myringotomy and tube insertion rates in New Brunswick, Canada. New Brunswick is a unique setting to study the impact of the Covid-19 pandemic because the province saw low case counts early in the pandemic, which resulted in less strain on the provincial healthcare system compared to other provinces, and subsequently less disruption of operating theatre functioning. Our data show a dramatic decrease in the number of bilateral myringotomy and tube insertions performed during the pandemic compared to before the pandemic. We interpret our data as indicating that public health measures used to control the spread of the pandemic are the most likely factor to explain this nearly threefold decrease in the number of bilateral myringotomy and tube insertions.

An alternate explanation for our results could have been a reduction in the number of otolaryngologists working in the province throughout the pandemic. In a small province such as New Brunswick, the departure or retirement of a small number of providers could significantly affect the data. However, we show that the number of surgeons performing paediatric bilateral myringotomy and tube insertions in New Brunswick was higher during the pandemic than before it (Figure 5). This was the result of otolaryngologists from Nova Scotia providing locum services in the underserviced northern part of New Brunswick and was unrelated to the pandemic.

We also considered that a reduction in the availability of operating theatre time might have been a significant factor in reducing the number of paediatric bilateral myringotomy and tube insertions during the pandemic. To address this possibility, we reviewed the number of total hip arthroplasties and cataract surgical procedures throughout the province over the same timeframe. We felt that these elective surgical procedures, which were performed by other specialists, would provide a good base for comparison of availability of operating theatre time, for both day of surgery admissions and for ambulatory procedures. Our data show that neither of these comparators saw a significant reduction in volume during the pandemic compared to pre-pandemic levels (Figure 4).

We also examined the rates of thyroid surgery as a comparator. Most otolaryngologists in New Brunswick carry out thyroid surgery, which is usually performed for cases of suspected or confirmed thyroid cancer. We wanted to assess whether urgent oncology thyroid surgery procedures were carried out more or less frequently than the other surgical procedures reviewed. We found that the number of thyroidectomies increased significantly during the pandemic (Figure 4d). We propose three potential explanations for this finding. Firstly, several of the new locum otolaryngologists providing services in northern New Brunswick were providing thyroid surgery. In the years prior to the pandemic, these cases were being referred to other centres within the province. This improved access likely led to higher numbers. Secondly, during the brief periods when operating theatres were partially restricted in the province, urgent cases were still being treated. In some centres, this resulted in more thyroid surgery procedures being conducted per month than usual, because they were some of the only cases otolaryngologists were permitted to book in. Lastly, the absolute number of thyroidectomies per month in the province is low, and therefore these data may be subject to type I (false-positive) errors.

The decreased rate of bilateral myringotomy and tube insertions during the pandemic is most likely due to public health measures aimed at reducing the transmission of respiratory viruses, including SARS-CoV-2. We propose that this led to fewer overall respiratory tract infections in children, leading to fewer instances of acute otitis media and otitis media with effusion, and thus fewer indications for paediatric bilateral myringotomy and tube insertions. New Brunswick acted early in the pandemic, promoting and eventually mandating masking and social distancing, and limiting close contacts.^5,6,11^ In addition, public health emphasised the importance of staying home when symptomatic, hand hygiene, sanitising surfaces and reducing contact with one's face. In the first year of the pandemic, the New Brunswick populace was generally compliant with these requests. Public health measures also focused on children by transitioning schools to virtual learning for the remainder of the 2020 academic year, temporarily closing day-care centres, and cancelling recreational sports and activities.^5^ Previous literature has noted that day-care and school attendance increases the incidence of URTIs in children.^12–14^ Studies also have shown reduced rates of otitis media and URTIs in children during the Covid-19 pandemic.^15–19^ Studies in the USA and Finland have also found lower rates of bilateral myringotomy and tube insertions during the Covid-19 pandemic.^20,21^ These findings support our hypothesis that the reduced rate of bilateral myringotomy and tube insertions in New Brunswick during the pandemic was due to decreased URTI transmission.

It has been suggested that a reluctance to seek healthcare during the pandemic, or an inability to access care, may have contributed to the reduced rate of paediatric bilateral myringotomy and tube insertions. Statistics Canada found that patients were more likely to delay seeking medical care in the first year of the pandemic due to perceptions of the healthcare system and Covid-19.^22^ Statistics Canada also noted that 4 out of 5 survey respondents had trouble accessing healthcare, and 1 out of 10 respondents needing healthcare could not schedule appointments during the first year of the pandemic.^22^ Other literature reported a decrease in emergency department visits, especially early in the pandemic.^15,23^ Some patients reported concerns regarding exposure to Covid-19 and unnecessarily burdening the healthcare system.^22^ All of these factors theoretically could have resulted in parents delaying appointments, not seeking care, or not being able to access care for their children who might have otherwise been offered bilateral myringotomy and tube insertions. However, the number of paediatric bilateral myringotomy and tube insertions performed in New Brunswick continued to decrease as the pandemic progressed, even as access to care improved, suggesting that these could not be the primary causes for the dramatic reduction we have reported (Figure 3). Furthermore, if an inability or reluctance to access care were the main drivers of our findings, we would have expected similar declines in the number of total hip arthroplasties, cataract surgical procedures and thyroidectomies throughout the study period, and our data show no commensurate reduction in those procedures.

- The coronavirus disease 2019 pandemic resulted in public health measures to reduce transmission of upper respiratory tract infections (URTIs)
- Paediatric bilateral myringotomy and tube insertion is often performed for indications secondary to URTI
- This study assessed the effect of these public health measures on the paediatric bilateral myringotomy and tube insertion rate
- The monthly paediatric bilateral myringotomy and tube insertion rate significantly decreased by 2.9 times during the pandemic
- This indicates the importance of maintaining appropriate masking, hand hygiene and staying home as the norm when symptomatic

The reduction in bilateral myringotomy and tube insertions, which has been an unexpected outcome of the Covid-19 pandemic, highlights the importance of making hand hygiene, appropriate masking and staying home when sick the norm. This would require a monumental cultural shift, but it has the potential to dramatically reduce the number of bilateral myringotomy and tube insertions operations performed. This in turn would reduce the number of children being exposed to the risks of a general anaesthetic, and bilateral myringotomy and tube insertion post-operative complications, and it could free up valuable operating theatre time.

The assessment of this topic is the first in Canada. By using the Medicare billing database as our data source, our study captured every bilateral myringotomy and tube insertions case performed in the entire province of New Brunswick over six years. All otolaryngologists in the province are remunerated through the fee-for-service payment model, so this database was judged to be the most accurate and accessible source of case counts in the province. However, the database lacks information regarding diagnoses and indications for surgery. These data, if gleaned through a more traditional chart review, potentially could have shed further light on the underlying reasons for the results we have shown. We attempted to mitigate this limitation by including only children in our study population. Given that the vast majority of bilateral myringotomy and tube insertions performed in children are for either recurrent acute otitis media, or persistent otitis media with effusion, both of which are strongly linked to respiratory tract infections, we did not feel that identifying the indications for surgery would have affected our results in any meaningful way.

Another limitation of our study is that it was not a randomised, controlled trial. Randomising a group of patients to strict public health restrictions, including mandatory masking, social distancing and staying home from school, would be impossible in our society. However, the climate of the pandemic created the conditions for this natural experiment, resulting in what we interpret to be the highest achievable level of evidence for this topic. Future research on the bilateral myringotomy and tube insertions rate after the lifting of public health measures would be interesting in order to assess whether this rate will return to pre-pandemic levels, which would further support our hypothesis.

## Conclusion

The rate of paediatric bilateral myringotomy and tube insertions in New Brunswick, Canada, during the Covid-19 pandemic significantly decreased by 2.9 times compared to pre-pandemic levels. This is likely due to public health measures targeting SARS-CoV-2 and reducing the transmission of respiratory viruses, resulting in decreased URTIs in children, and thus fewer indications for paediatric bilateral myringotomy and tube insertion. Our results show the value and importance of hand hygiene, appropriate masking, and staying home when sick, particularly amongst children.
